# Depression as a risk factor for dementia in older people with type 2 diabetes and the mediating effect of inflammation

**DOI:** 10.1007/s00125-020-05301-6

**Published:** 2020-10-16

**Authors:** Alistair L. Carr, Anniek J. Sluiman, Sheila M. Grecian, Rachel Forster, Stela McLachlan, Mark W. J. Strachan, Jackie F. Price

**Affiliations:** 1 Usher Institute, University of Edinburgh, Edinburgh, UK; 2 Metabolic Unit, Western General Hospital, Edinburgh, UK

**Keywords:** Cognition, Dementia, Depression, Inflammation, Older adults, Prospective, Type 2 diabetes

## Abstract

**Aims/hypothesis:**

We aimed to determine the association of depression with dementia risk in people with type 2 diabetes, and to explore the possible mediating role of inflammation in this relationship.

**Methods:**

The Edinburgh Type 2 Diabetes Study is a prospective cohort of 1066 men and women with type 2 diabetes aged 60–75 years. Cox proportional hazards regression analysis was used to investigate the association between depression, assessed at baseline, and subsequent risk of dementia over 10 years. Depression was defined using the Hospital Anxiety and Depression Scale, while incident dementia was defined using medical records, prescription data and death certificates. The potential mediating effect of systemic inflammation was assessed by adjusting models for a generalised inflammation factor, derived from four inflammatory markers measured at baseline (C-reactive protein, IL-6, TNF-α and fibrinogen), and carrying out an exploratory mediation analysis.

**Results:**

Dementia developed in 105 participants over a median follow-up of 10.6 years. After adjusting for age and sex, depression was associated with over a 2.5-fold increase in risk of dementia (HR 2.59 [95% CI 1.62, 4.15]). Additional adjustment for the generalised inflammation factor and other covariates did not attenuate the size of association between depression and incident dementia and mediation analysis showed that it was not a mediator. Adjusted logistic regression models showed cross-sectional associations of C-reactive protein and IL-6 with depression.

**Conclusions/interpretation:**

Depression is an important risk factor for dementia in people with type 2 diabetes. Some inflammatory markers were associated with depression, but systemic inflammation does not appear to mediate the relationship between depression and dementia.

**Figure:**
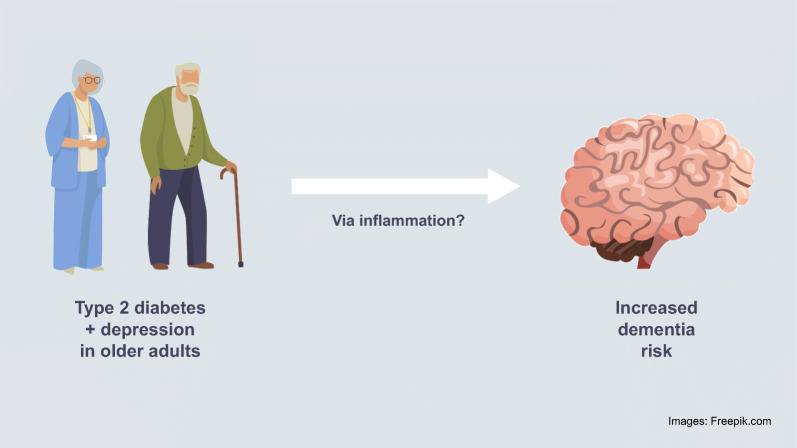
Graphical abstract

**Electronic supplementary material:**

The online version of this article (10.1007/s00125-020-05301-6) contains peer-reviewed but unedited supplementary material, which is available to authorized users.

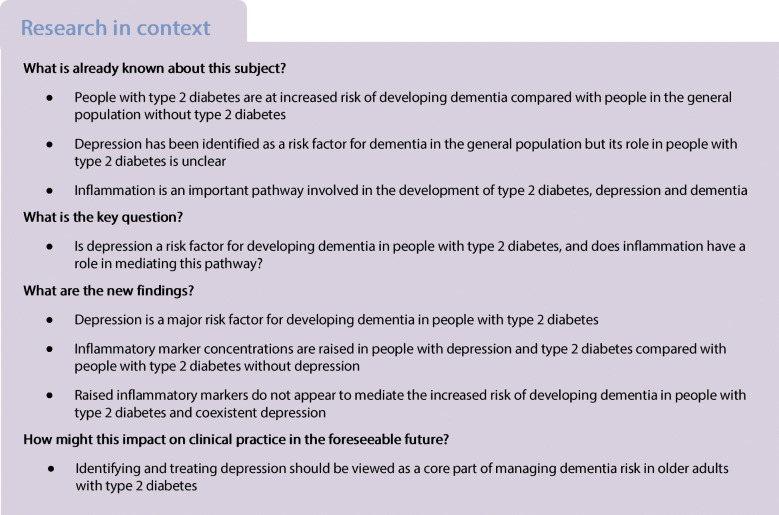



## Introduction

People with type 2 diabetes are 60% more likely to develop dementia compared with people in the general population [1]. Identifying and treating potentially modifiable risk factors in people with type 2 diabetes while they are cognitively healthy can help reduce cognitive morbidity in later life.

Late-life depression is a recognised risk factor for dementia in the general population [2]. In people with diabetes, a small number of studies with limited follow-up have also suggested depression is associated with increased risk of dementia [3–5]. It appears that in people with both diabetes and depression, the increased risk of dementia and cognitive decline is greater than the additive effect of the increased risk seen in people with only diabetes or only depression, suggesting the diseases may have a synergistic effect on dementia risk when they coexist [4, 6]. As depression is up to twice as common in people with type 2 diabetes than in the general population, this could have significant consequences from a public health perspective [7, 8].

The mechanisms that could explain the increase in dementia risk associated with coexistent depression and diabetes are unknown. In people with diabetes, depression is associated with increased prevalence of micro- and macrovascular disease [9, 10], poor glycaemic control [11] and unhealthy lifestyle behaviours such as physical inactivity, smoking and medication non-adherence [12]. However, after adjusting for these potential intermediate risk factors, people with both depression and diabetes still have approximately double the risk of dementia compared with people with only diabetes [3–5].

This increased risk of dementia could be due to inflammation, a biological mechanism important in the pathophysiologies of depression, type 2 diabetes and dementia. The presence of a sustained immune response is viewed as one of the main pathogenic mechanisms for both Alzheimer’s disease [13] and vascular dementia [14]. In the general population, associations have been found between concentrations of several inflammatory markers and increased risk of all-cause dementia [15]. Markers of inflammation have also been strongly associated with the presence and progression of depression [16], while anti-inflammatory medications may improve antidepressant treatment effects [17]. Raised circulating inflammatory markers have been associated with increased risk of type 2 diabetes [18], as well as with depressive symptoms [19] and cognitive decline [20, 21], in this group. Inflammatory mediators may therefore have an important role in the development of dementia in people with both depression and type 2 diabetes. This may be due to the hostile neural environment created when these conditions coexist, either via a direct effect of inflammatory markers on the brain, or indirectly through the development of vascular disease.

We conducted a prospective cohort study in an older cohort of people with type 2 diabetes to investigate the association between depression and dementia risk and the potential mediating role of inflammation.

## Methods

### Study population

Recruitment and examination in the Edinburgh Type 2 Diabetes Study (ET2DS) have been reported previously [22]. In brief, in 2006/2007 a cohort of 1066 men and women aged 60–75 years living with type 2 diabetes in the Lothian region of Scotland were selected from a population-based diabetes registry to attend baseline clinics where they underwent clinical assessment, including for diabetic retinopathy. Participants were invited to attend year 4 and year 10 follow-up clinics, and non-attenders were followed up through postal questionnaires and linkage to medical records. All participants gave written informed consent and ethical approval was granted by the Lothian Medical Research Ethics Committee.

### Depression

The self-completed Hospital Anxiety and Depression Scale (HADS) was used to measure depression status [23]. Compared with depression identified by clinical interview, a depression subscale (HADS-D) score ≥8 has a sensitivity of 0.82 and a specificity of 0.74, and a score ≥11 a sensitivity of 0.56 and specificity of 0.92, for detecting cases of major depressive disorder [24]. For the main analysis a baseline HADS-D score ≥8 was used to define depression. Sensitivity analyses were also performed using: (1) HADS-D score ≥11 (severe depression); (2) HADS-D score ≥8 at both baseline and year 4; and (3) either baseline HADS-D score ≥8 or an antidepressant prescription (fluoxetine hydrochloride, citalopram hydrochloride, paroxetine hydrochloride, sertraline hydrochloride, fluvoxamine maleate, escitalopram, isocarboxazid, moclobemide, mianserin, nefazodone hydrochloride, mirtazapine, venlafaxine, reboxetine, duloxetine hydrochloride or agomelatine). Tricyclic antidepressants were not included due to their frequent use for insomnia and neuropathic pain.

### Dementia

Incident all-cause dementia was identified through multiple sources: ICD-10 dementia codes from hospital admissions (1981 up to 2015, Scottish Morbidity Record-1 scheme); NHS Lothian electronic medical records system (Trakcare), which contained records of lifetime clinical dementia diagnosis (searched November 2017 to February 2018); death certificate records of dementia as primary or secondary cause of death (examined February 2018); and prescription data for dementia medication. A formal diagnosis of dementia required at least two of these criteria to be met or one of the criteria and one other indicator collected at the research clinics (year 4 or year 10 Mini Mental State Exam score ≤24, a self- or carer-reported dementia diagnosis or a GP-reported dementia diagnosis).

### Inflammatory markers

Venous blood samples were assayed for plasma C-reactive protein (CRP), IL-6, TNF-α and fibrinogen at the University Department of Medicine, Glasgow Royal Infirmary. CRP was assayed using a high-sensitivity immunonephelometric assay (detection limit 0.1 mg/l) and TNF-α (detection limit 0.1 pg/ml) and IL-6 antigen levels were assayed using high-sensitivity ELISA kits (R&D Systems, Oxon, UK). Left-censored values for CRP (*n* = 13) and TNF-α (*n* = 21) (below the minimum detection threshold) were replaced with values equal to half the minimum detected value. No values were right censored. Due to risk of acute infection, participants with CRP >10 mg/l were excluded from primary analyses involving inflammatory marker data but were included in a sensitivity analysis [25]. As the four inflammatory markers were highly correlated, we derived a single generalised inflammation factor based on the first principal component of a principal component analysis. For this, CRP, IL-6 and TNF-α were log-transformed as they had skewed distributions. The generalised inflammation factor explained 44.2% of the variance in the inflammatory marker data.

### Additional covariates

The presence of macrovascular disease was accepted if participants had a history of myocardial ischaemia, angina, stroke or transient ischaemic attack, as detailed previously [22]; hypertension was defined as systolic blood pressure >140 mmHg, diastolic blood pressure >90 mmHg or anti-hypertensive medication, and dyslipidaemia as total serum cholesterol >7.5 mmol/l or a lipid-lowering medication. Baseline prescription data were used to identify prescribed medicines that could potentially have anti-inflammatory effects (statins, fibrates, systemic steroids, non-steroidal anti-inflammatory medications and anti-thrombotic medications).

### Data analysis

Unadjusted statistical analyses were conducted using Student’s *t* test for normally distributed continuous data, the Mann–Whitney *U* test for non-normally distributed continuous data and the *χ*^2^ test for categorical data. Spearman’s rank correlation analysis was used to investigate the associations between inflammatory marker concentrations and continuous HADS-D score. Inflammatory markers with skewed distributions (CRP, IL-6 and TNF-α) were log-transformed and analysed using logistic regression models to assess the association between each inflammatory marker and depression status, adjusting for age, sex, prescribed medications and BMI. Cox proportional hazards (PH) models were used to investigate the association between depression and incident dementia. Participants contributed at-risk time from their baseline clinic date. Data were censored at dementia diagnosis, death or study endpoint (1 January 2018, the mid-point of the Trakcare search period). As Cox PH models require exact dates, but only the year of diagnosis was available for dementia diagnoses identified through Trakcare, 1 July was imputed as the mid-year point for Trakcare dementia diagnosis dates. Date of dementia diagnosis was then taken as the earliest date that dementia was identified from ICD-10 codes, Trakcare or death certificates. If the exact ICD-10 diagnosis date and Trakcare year of diagnosis were in the same year, the ICD-10 date was used. Multivariable Model 1 was adjusted for age and sex. Multivariable Model 2 was additionally adjusted for the generalised inflammation factor. Multivariable Model 3 was adjusted for: age, sex, marital status, education status, employment status, Scottish Index of Multiple Deprivation (SIMD), BMI, smoking, hypertension, dyslipidaemia, macrovascular disease, prescribed medications, diabetic retinopathy, diabetes duration, diabetes medication and HbA_1c_. Multivariable Model 4 included the same covariates as Multivariable Model 3 with the addition of the generalised inflammation factor. To minimise the risk of early-stage dementia being misdiagnosed as depression, sensitivity analyses were carried out where a dementia diagnosis time lag was applied, such that dementia cases diagnosed between baseline and year 2, and baseline and year 4, were excluded from the analysis.

### Mediation analysis

**Fig. 1:**
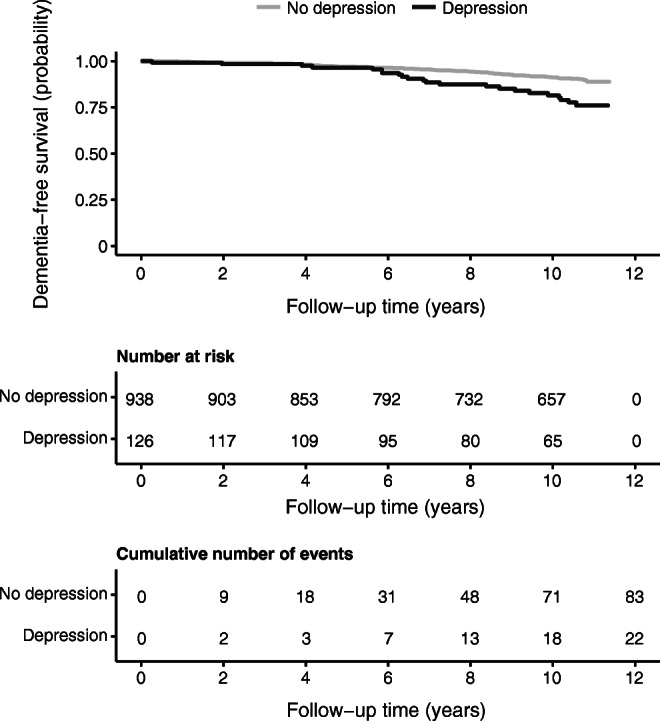
Kaplan–Meier curves of dementia-free survival probability by follow-up time, stratified by depression status (HADS-D≥8). Displayed below are the corresponding number at risk and cumulative events tables

The potential mediating role of inflammation was assessed using two methods. First, the causal steps method was used [26]. In this method, *X* (depression) indicates the independent variable, *Y* (dementia) indicates the dependent variable and *M* (generalised inflammation factor) indicates the mediator (see electronic supplementary material [ESM] Fig. 1). Partial mediation is considered to have occurred if: (1) *X* is related to *Y*; (2) *X* is related to *M*; (3) *M* is related to *Y* after adjusting for *X*; and (4) the association between *X* and *Y* is significantly decreased after adjusting for *M*. As the dementia variable contained censored data, accelerated failure time models were used to assess the relationship between: (1) depression and dementia; and (2) inflammation and dementia [27], and a linear regression model was used to assess the effect of depression on inflammation. Second, mediation was assessed using the bootstrapping method so that the percentage of the association between depression and dementia mediated by the generalised inflammation factor could be estimated [28].

### Competing risk of death

Due to the advanced age of the cohort, large numbers of participants may have died before dementia could be diagnosed. As competing risk of death is not accounted for in standard Cox PH models, this has the potential to lead to an overestimation of the effect size [29]. The effect of this was investigated with a competing risk regression (CRR) model [30].

## Results

### Baseline characteristics

A total of 1066 people were enrolled in the ET2DS. Of these, two participants were excluded from further analysis; one as they had dementia at baseline (applying the same criteria as used during follow-up) and the second as illness prevented them from completing the HADS. Of the remaining 1064 participants, 62 had CRP concentrations >10 mg/l that were excluded from analyses involving inflammatory marker data. Depression (HADS-D ≥8) was present in 126 participants and severe depression (HADS-D ≥11) in 28. Participants with depression were more likely to be female, to be single or widowed, and to live in the most deprived quintile of socioeconomic deprivation (Table 1). They were also more likely to have a higher BMI, to smoke, to have macrovascular disease, to manage their diabetes with insulin and to have a higher mean HbA_1c_. Anxiety (HADS anxiety subscale [HADS-A] ≥ 8) was present in 99 participants (78.6%) with depression.

**Table 1:** Baseline characteristics of study participants by depression status

Variable	Total cohort (*N* = 1064)	With depression (*n* = 126)^a^	Without depression (*n* = 938)	*p* value
Mean age, years (SD)	67.9 (4.2)	67.4 (4.2)	68.0 (4.2)	0.170
Sex, *n* (%)				
Male	546 (51.3)	50 (39.7)	496 (52.9)	0.005
Female	518 (48.7)	76 (60.3)	442 (47.1)	
Marital status, *n* (%)				
Married/living with long-term partner	796 (75.0)	84 (66.7)	712 (76.1)	0.036
Single	159 (15.0)	22 (17.5)	137 (14.6)	
Widowed	107 (10.1)	20 (15.9)	87 (9.3)	
Highest level of educational attainment, *n* (%)				
University/college	171 (16.1)	16 (12.7)	155 (16.5)	0.196
Other professional/technical qualification	305 (28.7)	31 (24.6)	274 (29.2)	
Primary/secondary school	588 (55.3)	79 (62.7)	509 (54.3)	
Current employment status, *n* (%)				
Full/part-time employment	152 (14.3)	10 (7.9)	142 (15.1)	0.047
Retired	862 (81.0)	107 (84.9)	755 (80.5)	
Unemployed/homemaker/other	50 (4.7)	9 (7.1)	41 (4.4)	
SIMD quintile, *n* (%)				
First (most deprived)	127 (11.9)	24 (19.0)	103 (11.0)	0.086
Second	207 (19.5)	23 (18.3)	184 (19.6)	
Third	188 (17.7)	21 (16.7)	167 (17.8)	
Fourth	194 (18.2)	21 (16.7)	173 (18.4)	
Fifth (least deprived)	348 (32.7)	37 (29.4)	311 (33.2)	
HADS-A score ≥8, *n* (%)				
Yes	321 (30.2)	99 (78.6)	222 (23.7)	<0.001
No	743 (69.8)	27 (21.4)	716 (76.3)	
Median HADS-A score (IQR)	5.0 (5.0)	10.0 (5.0)	5.0 (4.0)	<0.001
Median HADS-D score (IQR)	3.0 (5.0)	9.0 (2.0)	3.0 (4.0)	<0.001
Mean BMI (SD)	31.4 (5.7)	33.3 (6.6)	31.2 (5.5)	<0.001
Smoking status, *n* (%)				
Smoker	153 (14.4)	27 (21.4)	126 (13.4)	0.016
Non-smoker/ex-smoker	911 (85.6)	99 (78.6)	812 (86.6)	
Hypertension, *n* (%)				
Yes	917 (86.3)	111 (88.1)	806 (86.1)	0.543
No	145 (13.7)	15 (11.9)	130 (13.9)	
Dyslipidaemia, *n* (%)				
Yes	913 (86.1)	110 (88.0)	803 (85.8)	0.503
No	148 (13.9)	15 (12.0)	133 (14.2)	
Macrovascular disease, *n* (%)				
Yes	373 (35.1)	63 (50.0)	310 (33.0)	<0.001
No	691 (64.9)	63 (50.0)	628 (67.0)	
Diabetic retinopathy, *n* (%)				
Yes	339 (32.5)	43 (34.7)	296 (32.2)	0.582
No	704 (67.5)	81 (65.3)	623 (67.8)	
Median time since diabetes diagnosis, years (IQR)	6.0 (8.0)	7.0 (8.0)	6.0 (8.0)	0.784
Diabetes treatment, *n* (%)				
Diet only	198 (18.6)	23 (18.3)	175 (18.7)	0.009
Diet + oral tablets	680 (64.0)	69 (54.8)	611 (65.2)	
Diet ± oral tablets + insulin	185 (17.4)	34 (27.0)	151 (16.1)	
Mean HbA_1c_, mmol/mol (SD)	57.5 (12.3)	59.8 (14.7)	57.2 (11.9)	0.027
Mean HbA_1c_, % (SD)	7.4 (1.1)	7.6 (1.3)	7.4 (1.1)	0.027
Mean fibrinogen, g/l (SD)	3.6 (0.7)	3.7 (0.7)	3.6 (0.7)	0.009
Median IL-6, pg/ml (IQR)	2.8 (2.3)	3.4 (2.6)	2.7 (2.2)	0.001
Median TNF-α, pg/ml (IQR)	1.1 (0.9)	1.2 (0.9)	1.1 (0.9)	0.258
Median CRP, mg/l (IQR)	1.7 (2.8)	2.8 (5.2)	1.6 (2.6)	<0.001
Mean generalised inflammation factor (SD)	0.0 (1.3)	0.5 (1.3)	−0.1 (1.3)	<0.001

*p* values calculated by *χ*^2^ test for categorical variables, Student’s *t* test for normally distributed continuous data and Mann–Whitney *U* test for non-normally distributed continuous data, comparing the groups with and without depression. Missing or incomplete values were as follows: CRP=24 cases; TNF-α=3 cases; fibrinogen=3 cases; IL-6=2 cases; diabetic retinopathy=21 cases; diabetes duration=13 cases; HbA_1c_=9 cases; BMI=3 cases; marital status=2 cases; hypertension status=2 cases; dyslipidaemia status=2 cases

^a^HADS-D score ≥8

### Associations of inflammatory mediators with depression at baseline

Spearman’s Rank correlation analysis showed weak but statistically significant associations with all inflammatory marker concentrations and continuous HADS-D score (Table 2). Of the 1002 participants with non-raised CRP concentrations, 116 had depression and 24 severe depression. Univariate logistic regression models, where depression status was the dependent variable, showed statistically significant associations with fibrinogen, IL-6, CRP and the generalised inflammation factor, but not with TNF-α. When the models were adjusted for age, sex, BMI and medications, the association of fibrinogen with depression was no longer statistically significant. When severe depression was used as the dependent variable and the same covariates were adjusted for, a statistically significant association was found with CRP (OR 2.21 [95% CI 1.29, 4.05]) and the generalised inflammation factor (OR 1.57 [95% CI 1.11, 2.23]), but not with fibrinogen (OR 1.34 [95% CI 0.73, 2.44]), IL-6 (OR 1.74 [95% CI 0.90, 3.25]) or TNF-α (OR 1.60 [95% CI 0.91, 2.82]).

**Table 2:** Association between inflammatory markers and depression

Inflammatory marker	Spearman’s Rank correlation coefficient^a^, *ρ*	Unadjusted logistic regression model^b^, OR (95% CI)	Adjusted logistic regression model^c^, OR (95% CI)
Fibrinogen	0.109 (*p* < 0.001)	1.45 (1.09, 1.92)	1.33 (0.99, 1.79)
IL-6	0.147 (*p* < 0.001)	1.59 (1.19, 2.11)	1.59 (1.17, 2.15)
TNF-α	0.067 (*p* = 0.034)	1.13 (0.89, 1.46)	1.11 (0.86, 1.44)
CRP	0.161 (*p* < 0.001)	1.58 (1.29, 1.97)	1.59 (1.17, 2.15)
Generalised inflammation factor	0.176 (*p* < 0.001)	1.38 (1.19, 1.61)	1.35 (1.15, 1.59)

^a^Association between inflammatory marker concentration and continuous HADS-D score

^b^Association between inflammatory marker concentration and depression (HADS-D≥8)

^c^Association between inflammatory marker concentration and depression (HADS-D≥8). Adjusted for age, sex, BMI, lipid-lowering medications, non-steroidal anti-inflammatory drugs (NSAIDs), anti-thrombotic medication and systemic steroids

### Dementia incidence, association with depression and possible mediation by inflammation

Over a median follow-up of 10.6 years (IQR 8.4–11.0), 105 participants developed dementia. Participants who developed dementia were older (69.4 vs 67.7 years), more likely to be male (61% vs 50.3% male) and more likely to have depression (21.0% vs 10.8%) compared with those without dementia. Diabetes treatment and glycaemic control were similar across both groups, but those with dementia had a higher prevalence of diabetic retinopathy (42.2% vs 31.5%) and macrovascular disease (42.9% vs 34.2%) (ESM Table 1). Most cases of dementia were diagnosed in the depression group from around year 6 of follow-up onwards (Fig. 1). During follow-up there were 317 deaths, and, of these, 41 deaths were in participants that had developed dementia. The age- and sex-adjusted Cox PH model showed that depression was associated with over a 2.5-fold increased risk of dementia (HR 2.59 [95% CI 1.62, 4.15]). Additional adjustment for the generalised inflammation factor and other covariates did not significantly change the effect size (Table 3). In sensitivity analyses, changing the depression case definitions also did not substantially change the observed effect (ESM Table 2), including when the 2 year and 4 year dementia diagnosis time lags were applied. The age- and sex-adjusted HR when the independent variable was severe depression was 4.40 (95% CI 1.88, 10.26), and 4.39 (95% CI 2.37, 8.15) when it was depression, at baseline and year 4.

**Table 3:** Association between baseline depression and incident dementia

Model	HR (95% CI)
Unadjusted	2.35 (1.47, 3.77)
Multivariable Model 1^a^	2.59 (1.62, 4.15)
Multivariable Model 2^b^	2.34 (1.41, 3.89)
Multivariable Model 3^c^	2.54 (1.51, 4.26)
Multivariable Model 4^d^	2.37 (1.37, 4.10)

^a^Adjusted for age and sex; number in model = 1064; number of events=105

^b^Adjusted for age, sex and generalised inflammation factor; number in model=1002; number of events=99

^c^Adjusted for age, sex, marital status, education status, employment status, SIMD, BMI, smoking, hypertension, dyslipidaemia, macrovascular disease, prescribed medications, diabetic retinopathy, diabetes duration, diabetes medication and HbA_1c_; number in model=114; number of events=99

^d^Adjusted for age, sex, marital status, education status, employment status, SIMD, BMI, smoking, hypertension, dyslipidaemia, macrovascular disease, prescribed medications, diabetic retinopathy, diabetes duration, diabetes medication, HbA_1c_ and generalised inflammation factor; number in model=955; number of events=93

### Mediation analysis

The causal steps regression analyses showed that depression was positively associated with higher levels of inflammation (*β*: 0.57, *p* < 0.001) (ESM Fig. 1). Depression was associated with having a 0.65 times shorter time period before developing dementia without adjusting for inflammation (*p* < 0.01), and a 0.66 times shorter time period when inflammation was adjusted for (*p* < 0.01). However, the association between inflammation and dementia was non-significant when depression was adjusted for (*p* = 0.777), indicating that inflammation did not mediate the association between depression and dementia. Mediation analysis using bootstrapping with 5000 replications also showed that inflammation was not a mediator, accounting for only 1.7% (95% CI −13, 19) of the association between depression and dementia (ESM Table 3).

### Competing risk analysis

The age- and sex-adjusted CRR model showed that depression had a similar effect size (HR 2.24 [95% CI 1.40, 3.60]) to dementia risk when compared with the standard Cox PH model.

### Sensitivity analyses

In total, 49 participants were prescribed an antidepressant. Of these, 16 had a HADS-D score ≥8 and 33 a HADS-D score <8. Incorporating the antidepressant data into the depression case definition therefore increased the number of cases to 159. Re-analysing the data using this definition of depression did not change any of the main findings, nor did including the inflammatory marker data from the 62 participants that had a raised CRP (data not shown).

## Discussion

### Principal findings

The prospective association between depression and dementia was investigated in a cohort of older people with type 2 diabetes. We found that depression in later life, after adjusting for age and sex, was associated with over a 2.5-fold increase in dementia risk. The relationship remained constant in a series of sensitivity analyses where different criteria were used to define depression. Cross-sectional associations of inflammatory markers with depression status were tested and showed positive associations with IL-6 and CRP. To explore whether systemic inflammation had a role as a mediator in the relationship between depression and dementia, models were adjusted for a generalised inflammation factor, derived from four inflammatory markers measured at baseline, and a mediation analysis was undertaken. Adjusting for the generalised inflammation factor did not substantially change the effect size between depression and dementia, and mediation analysis found no evidence of mediation. Adjusting for other potentially important risk factors, including smoking, obesity, hypertension, vascular disease and HbA_1c_, also did not substantially alter the effect size between depression and dementia.

### Strengths and weaknesses

A strength of this study was that we were able to use a well-characterised cohort of participants with type 2 diabetes that had inflammatory markers measured at baseline. The cohort was also relatively homogenous in terms of age, ethnicity and glycaemic control, meaning these potential confounders were unlikely to influence our results. Another strength was that through using multiple data collection methods we were able to follow up our cohort prospectively for 10 years with minimal loss to follow-up. The study also had a number of limitations. First, our method of identifying dementia relied on the analysis of collected medical data which were reliant on accurate clinical diagnosis and reporting. Some participants with dementia may have accessed health care infrequently and some may have died before a diagnosis could be made. The effect of competing risk of death was investigated with a CRR analysis, which showed that the effect of this was likely to be small. Furthermore, due to their similar clinical presentations, some clinicians may have incorrectly diagnosed depression as dementia. The same problem may have resulted in some cases of early-stage dementia being misdiagnosed as depression. This is further complicated by evidence that suggests late-life depression can be a prodrome of dementia for several years before dementia develops [31]. As the effect of depression on incident dementia in our cohort became apparent after year 6 of follow-up, it appears likely that we were successful in being able to separately identify cases of depression and dementia. Another limitation of our depression definition was that it was only measured at baseline so the effect of duration of depressive symptoms could not be evaluated. To attempt to address these issues, sensitivity analyses were carried out where the time between depression diagnosis and dementia was increased to minimise the risk of dementia being misdiagnosed as depression, and different depression case definitions were used that incorporated antidepressant medications and depression at year 4. Ideally, depression would have been identified by clinical interview, earlier in the life course and measured repeatedly. And, ideally, repeated measurements of inflammatory markers would also have been incorporated into the analysis, rather than measurements from a single time point as used in this analysis, to more effectively identify participants with long-term increased levels of systemic inflammation. Finally, although associations were adjusted for a wide range of important risk factors, the independence of the relationships from additional potential risk factors for dementia in people with type 2 diabetes, for example, genetic susceptibility, anxiety and hypoglycaemia, would require further in-depth analysis. The generalisability of our study is limited due to the study population being derived from a single geographical region with access to a well-developed national healthcare system.

### Comparison with other studies

Depression is a recognised risk factor for dementia in the general population [2, 31], but in studies of cohorts exclusively comprising of people with diabetes, a group at higher risk of dementia than the general population, the effect of depression on dementia risk has only been investigated in two 5 year prospective cohort studies and one large retrospective study, which all have the same authorship [3–5]. Each study found coexistent depression in people with diabetes to be associated with an approximately twofold increase in dementia risk. The larger effect size found in the present study may be related to the longer period of follow-up. In keeping with the findings of the previous studies, no attenuation in effect size was found after adjusting for important clinical and social covariates. Prior studies were unable to include adjustment for inflammation, which has been suggested as a biological mechanism that could mediate the development of dementia in people with both type 2 diabetes and depression [3–5, 32]. We did not find any evidence to support this hypothesis. This is in contrast to a cross-sectional study that investigated the association of inflammatory markers with mild cognitive impairment (MCI) in people with type 2 diabetes and coexisting depression. It found CRP, IL-6 and TNF-α to be associated with increased risk of MCI [21]. We found cross-sectional associations of depression status with raised inflammatory marker concentrations. When assessed as a continuous variable, associations were found between HADS-D score and all measured inflammatory markers, but when analysed as a binary variable in adjusted models, associations were only found with CRP and IL-6. This is partially in keeping with the results of prior studies, which are low in number and mostly have small sample sizes [19]. The most commonly measured inflammatory marker has been CRP, which, in keeping with our findings, has been found to be associated with depression in people with both type 2 diabetes and depression [33–36]. Only one prior study that measured IL-6 found an association with IL-6 and depression in people with type 2 diabetes [33], whereas two studies did not [34, 36]. Two studies, one of which consisted of over 1200 adults with newly diagnosed type 2 diabetes, measured TNF-α concentrations [33, 34]. Both, in keeping with the results of our adjusted logistic regression analysis, did not find an association between TNF-α and depression. Only one study measured fibrinogen and found it to be raised in people with both type 2 diabetes and depression compared with those with diabetes alone [37]. Of note, different depression scoring systems were used between studies and this has been shown previously to affect associations found with specific inflammatory markers [38].

### Study meaning and implications

In people with type 2 diabetes, a single episode of depressive symptoms in later life places them at high risk of later developing dementia. It appears that this relationship is not due to unhealthy lifestyle behaviours, diabetic severity or cardiovascular comorbidity, but rather a feature of the presence of depressive symptoms. Sensitivity analyses suggested that depression severity and duration may be associated with higher dementia risk but, as one would expect given the smaller sample sizes used for these analyses, the confidence intervals were wide and overlapped with those of the primary analysis, so this has to be interpreted with caution. We did not find any evidence to suggest that inflammation, as measured by a derived generalised inflammation factor, was responsible for mediating this relationship. The relationship could be explained by hypothalamic–pituitary–adrenal axis dysregulation and increased insulin resistance, both of which are pathways associated with depression and have been linked to the development of cognitive impairment [39, 40]. The association may also be partially explained by anxiety, which was prevalent in our cohort and has been shown to be a risk factor for dementia in the general population [41]. As a potentially modifiable risk factor, clinicians should regard effectively screening for, diagnosing and treating depression as a mainstay of the management of dementia risk in people that have type 2 diabetes. Our study shows that the HADS questionnaire may be a useful screening tool in this population. Our study showed that some inflammatory markers, especially CRP and IL-6, appeared to be associated with depression status. This could be due to both depression and diabetes being inflammatory conditions and may explain some of the increased risk of complications other than dementia for people with diabetes and comorbid depression.

### Unanswered questions and future research

As the burden of type 2 diabetes and depression on individuals and society continues to grow, further research is required to investigate the pathophysiological pathways that link these diseases with each other and with dementia. This will require the temporal association of the diseases to be evaluated in studies than can incorporate repeated measurements of depression, diabetes and dementia across the life course. Although our results suggest that inflammation is not an important part of this pathway, with only four inflammatory markers measured at one time point, analyses involving serial measurements of inflammatory markers are required. The prognostic significance of depression severity and duration on dementia risk, and the relationship of depression with anxiety, also requires further evaluation. The significance of raised CRP and IL-6 in people with both depression and type 2 diabetes is unclear and may be important in the development of other diabetes-related complications.

### Conclusion

We conclude that in a representative cohort of older people with type 2 diabetes, depression was associated with increased subsequent risk of developing dementia. As a potentially modifiable risk factor, treating depression should be considered a high priority in the management of dementia risk in people with type 2 diabetes. Our findings do not support the hypothesis that systemic inflammation is an important pathway that mediates this association, and further work is required to determine the underlying pathophysiological pathways.

## Electronic supplementary material

ESM (PDF 103 kb)

## Data Availability

The dataset analysed during the current study is available from the corresponding author on reasonable request.

## Abbreviations

CRP: C-reactive protein
CRR: Competing risk regression
ET2DS: Edinburgh Type 2 Diabetes Study
HADS: Hospital Anxiety and Depression Scale
HADS-A: HADS anxiety subscale
HADS-D: HADS depression subscale
MCI: Mild cognitive impairment
PH: Proportional hazards
SIMD: Scottish Index of Multiple Deprivation
