# Supplementary material for: Depression as a risk factor for dementia in older people with type 2 diabetes and the mediating effect of inflammation
Source: Diabetologia. 2020 Oct 16;64(2):448–57. doi: 10.1007/s00125-020-05301-6 (PMC7801357; doi:10.1007/s00125-020-05301-6)
Supplement: Supplementary file 1 — (PDF 103 kb) [file 125_2020_5301_MOESM1_ESM.pdf]

# Electronic Supplementary Material

## Tables

### ESM Table 1   Baseline characteristics of participants by dementia status

| Variable | Without dementia (*n*=959) | With dementia (*n*=105) |
|---|---|---|
| Mean age, years (SD) | 67.7 (4.2) | 69.4 (4.0) |
| Sex, *n* (%) | | |
| Male | 482 (50.3) | 64 (61.0) |
| Female | 477 (49.7) | 41 (39.0) |
| Marital status, *n* (%) | | |
| Married/living with long-term partner | 718 (74.9) | 78 (75.0) |
| Single | 144 (15.0) | 15 (14.4) |
| Widowed | 96 (10.0) | 11 (10.6) |
| Highest level of educational attainment, *n* (%) | | |
| University/college | 153 (16.0) | 18 (17.1) |
| Other professional/technical qualification | 285 (29.7) | 20 (19.0) |
| Primary/secondary school | 521 (54.3) | 67 (63.8) |
| Current employment status, *n* (%) | | |
| Full/part-time employment | 139 (14.5) | 13 (12.4) |
| Retired | 771 (80.4) | 91 (86.7) |
| Unemployed/homemaker/other | 49 (5.1) | 1 (1.0) |
| SIMD quintile, *n* (%) | | |
| First (most deprived) | 114 (11.9) | 13 (12.4) |
| Second | 190 (19.8) | 17 (16.2) |
| Third | 168 (17.5) | 20 (19.0) |
| Fourth | 167 (17.4) | 27 (25.7) |
| Fifth (least deprived) | 320 (33.4) | 28 (26.7) |
| HADS-A score ≥8, *n* (%) | | |
| Yes | 280 (29.2) | 41 (39.0) |
| No | 679 (70.8) | 64 (61.0) |
| Median HADS-A score (IQR) | 5.0 (5.0) | 5.0 (7.0) |
| HADS-D score ≥8, *n* (%) | | |
| Yes | 104 (10.8) | 22 (21.0) |
| No | 855 (89.2) | 83 (79.0) |
| Median HADS-D score (IQR) | 3.0 (5.0) | 4.0 (5.0) |
| Mean BMI (SD) | 31.6 (5.7) | 30.0 (5.2) |
| Smoking status, *n* (%) | | |
| Smoker | 143 (14.9) | 10 (9.5) |
| Non-smoker/ex-smoker | 816 (85.1) | 95 (90.5) |
| Hypertension, *n* (%) | | |
| Yes | 831 (86.8) | 86 (81.9) |
| No | 126 (13.2) | 19 (18.1) |
| Dyslipidaemia, *n* (%) | | |

| | | |
|---|---|---|
| Yes | 822 (86.0) | 91 (86.7) |
| No | 134 (14.0) | 14 (13.3) |
| Macrovascular disease, *n* (%) | | |
| Yes | 328 (34.2) | 45 (42.9) |
| No | 631 (65.8) | 60 (57.1) |
| Diabetic retinopathy, *n* (%) | | |
| Yes | 296 (31.5) | 43 (42.2) |
| No | 645 (68.5) | 59 (57.8) |
| Median time since diabetes diagnosis, years (IQR) | 6.0 (8.0) | 7.0 (9.2) |
| Diabetes treatment, *n* (%) | | |
| Diet only | 180 (18.8) | 18 (17.1) |
| Diet + oral tablets | 615 (64.2) | 65 (61.9) |
| Diet ± oral tablets + insulin | 163 (17.0) | 22 (21.0) |
| Mean $HbA_{1c}$, mmol/mol (SD) | 57.3 (11.7) | 58.9 (16.9) |
| Mean $HbA_{1c}$, % (SD) | 7.4 (1.1) | 7.5 (1.5) |

**ESM Table 2** Sensitivity analyses exploring the association between depression and incident dementia

| Model | Unadjusted HR (95% CI) | Age- and sex-adjusted HR (95% CI) |
|---|---|---|
| 1[a] | 2.31 (1.49, 3.59) | 2.60 (1.68, 4.05) |
| 2[b] | 2.76 (1.21, 6.29) | 4.40 (1.88, 10.26) |
| 3[c] | 2.45 (1.49, 4.01) | 2.67 (1.63, 4.39) |
| 4[d] | 2.70 (1.62, 4.50) | 2.95 (1.76, 4.92) |
| 5[e] | 3.77 (2.04, 6.97) | 4.39 (2.37, 8.15) |

[a]Depression defined by either baseline HADS-D score≥8 or at least one antidepressant prescription (*n*=159), number in model=1064, number of events=105

[b]Depression defined by baseline HADS-D score≥11 (*n*=28), number in model=1064, number of events=105

[c]Depression defined by baseline HADS-D score≥8 (*n*=126), with cases excluded where dementia was diagnosed during first 2 years of follow-up, number in model=1053, number of events=94

[d]Depression defined by baseline HADS-D score≥8 (*n*=126), with cases excluded where dementia was diagnosed during first 4 years of follow-up, number in model=1043, number of events=84.

[e]Depression defined by baseline and year-4 HADS-D score≥8 (*n*=51), with cases excluded where dementia was diagnosed during first 4 years of follow-up, number in model=1007, number of events=82.

**ESM Table 3** Direct and indirect effect of depression with dementia

| Effect | Association | |
|---|---|---|
| | $\beta$ (95% CI) | Proportion mediated, % (95% CI) |
| Total | -12.8 ( -22.3, -4.0) | |
| Direct | -12.6 (-22.3, -3.73) | |
| Indirect via the generalised inflammation factor | -0.21 (-1.82, 1.32) | 1.7 (-13, 19) |

Bootstrapping mediation analysis with 5,000 replications. Total effect is the effect of depression on dementia without generalised inflammation factor. Direct effect is the effect of depression on dementia when controlling for generalised inflammation factor. Indirect effect is the effect of depression on dementia via generalised inflammation factor. Proportion mediated (%) is the proportion of the effect of depression on dementia that goes via generalised inflammation factor (calculated by indirect effect/total effect x 100).

## Figures

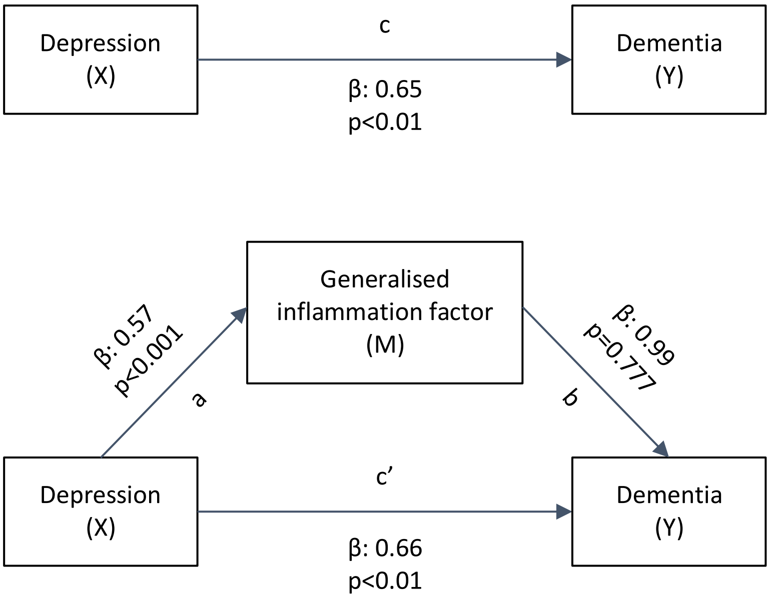


**ESM Fig. 1** Mediation analysis

The figure depicts the relationship between depression, dementia and the generalise inflammation factor. β: regression coefficient; X: independent variable; Y: dependent variable; M: mediator. Path a: X is related to M; path b: M is related to Y after adjusting for X; path c: X is related to Y; path c': X is related to Y when M is included in the model as a covariate. Paths b, c and c' were analysed using accelerated failure time models (number in models=1002) and the exponentiated beta coefficients of these models are displayed. Path a was analysed with a linear regression model (number in model=1002).
